# An Amphibious Fully‐Soft Centimeter‐Scale Miniature Crawling Robot Powered by Electrohydraulic Fluid Kinetic Energy

**DOI:** 10.1002/advs.202308033

**Published:** 2024-02-01

**Authors:** Quan Xiong, Xuanyi Zhou, Dannuo Li, Jonathan William Ambrose, Raye Chen‐Hua Yeow

**Affiliations:** ^1^ Department of Biomedical Engineering National University of Singapore 15 Kent Ridge Cres Singapore 119276 Singapore

**Keywords:** amphibious robots, design optimization, electrohydraulic actuators, reconfigurable robots, soft crawling robots, underwater actuators

## Abstract

Miniature locomotion robots with the ability to navigate confined environments show great promise for a wide range of tasks, including search and rescue operations. Soft miniature locomotion robots, as a burgeoning field, have attracted significant research interest due to their exceptional terrain adaptability and safety features. Here, a fully‐soft centimeter‐scale miniature crawling robot directly powered by fluid kinetic energy generated by an electrohydraulic actuator is introduced. Through optimization of the operating voltage and design parameters, the average crawling velocity of the robot is dramatically enhanced, reaching 16 mm s^−1^. The optimized robot weighs 6.3 g and measures 5 cm in length, 5 cm in width, and 6 mm in height. By combining two robots in parallel, the robot can achieve a turning rate of ≈3° s^−1^. Additionally, by reconfiguring the distribution of electrodes in the electrohydraulic actuator, the robot can achieve 2 degrees‐of‐freedom translational motion, improving its maneuverability in narrow spaces. Finally, the use of a soft water‐proof skin is demonstrated for underwater locomotion and actuation. In comparison with other soft miniature crawling robots, this robot with full softness can achieve relatively high crawling velocity as well as increased robustness and recovery.

## Graphical abstract

This paper presents a breakthrough in miniature crawling robots designed for confined environments, particularly beneficial for search and rescue. The fully‐soft centimeter‐scale robot, powered by fluid‐kinetic‐energy, achieves a remarkable crawling velocity of 16 mm s^−1^. The robot's soft, water‐proof skin further enables underwater locomotion, distinguishing it for its high crawling speed, robustness, and recovery compared to other soft miniature crawling robots.

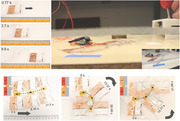

## 1 Introduction

Miniature locomotion robots operating at the micro‐ to centimeter length scales demonstrate remarkable locomotion and navigation capabilities within highly confined and unstructured spaces.^[^ ^1^, ^2^, ^3^, ^4^ ^]^ Their unique abilities have unlocked tremendous potential in various tasks, such as drug delivery, collection, search, and rescue operations.^[^ ^5^, ^6^ ^]^ Over the past few years, there has been a significant surge in research interest surrounding soft locomotion robots, primarily due to their exceptional terrain adaptability and inherent safety features.^[^ ^7^, ^8^, ^9^, ^10^, ^11^ ^]^ These robots are built with components made from deformable and compliant materials,^[^ ^12^, ^13^, ^14^ ^]^ allowing them to accommodate diverse surroundings and interact safely with uncertain environments.

Actuators play a critical role in determining the softness and performance of miniature robots. Generally, high bandwidth and power‐weight ratio are essential requirements for actuators in miniature locomotion robots. While some traditional rigid actuators, such as vibrating motors and piezoelectric actuators, easily meet these requirements,^[^ ^15^, ^16^, ^17^ ^]^ conventional soft actuators face significant challenges. Pneumatic actuators, as the predominant choice for soft actuators, have been widely utilized in soft miniature locomotion robots due to their simple structure and fabrication process.^[^ ^18^, ^19^, ^20^ ^]^ Nevertheless, their bandwidth is limited^[^ ^21^, ^22^ ^]^ (typically <5 Hz), and the dimension and weight of air tubes impede their miniaturization and hinder long‐distance locomotion. In response to these limitations, alternative soft actuators, such as dielectric elastomer actuators (DEAs) and shape memory alloys (SMAs), have been proposed. DEA‐based soft miniature robots exhibit high maneuverability.^[^ ^23^, ^24^, ^25^ ^]^ However, their fabrication is complex due to the pre‐stretching frame mechanism and the challenges posed by the miniature scale, and the risk of an electric breakdown may result in severe damage to the robot.^[^ ^26^, ^27^ ^]^ On the other hand, SMA actuators have a slow dynamic response due to the long cooling and recovery time required by the alloy coil to return to its initial state, thereby limiting the robot's overall performance.^[^ ^28^, ^29^ ^]^

The emerging soft electrohydraulic actuator, known as the hydraulically amplified self‐healing electrostatic (HASEL) actuator,^[^ ^30^ ^]^ exhibits exceptional characteristics, including rapid response and a high power‐weight ratio.^[^ ^31^ ^]^ Electrohydraulic actuators harness the Maxwell stress^[^ ^32^ ^]^ generated by electrostatic fields to compress the dielectric fluid within a bladder, utilizing the resulting deformation of the bladder as a source of mechanical actuation. This bladder deformation mechanism of electrohydraulic actuators has found widespread application in various robotic systems, including robotic grippers,^[^ ^33^, ^34^, ^35^ ^]^ locomotion robots,^[^ ^36^, ^37^ ^]^ and artificial muscles.^[^ ^31^, ^38^ ^]^ However, the investigation and application on the directly‐generated kinetic energy of the dielectric fluid, which arises from its compression by the Maxwell stress, remains relatively limited and unexplored.

In this study, we present a fully‐soft miniature crawling robot (MCR) directly powered by electrohydraulic fluid kinetic energy (EFKE). The MCR here consists of only a single soft body that is lightweight and compact. We use an electrohydraulic actuator to generate unidirectional EFKE, such that its liquid inertia propels the MCR along the ground. To extend the robot's mobility to underwater environment, we have designed a soft water‐proof skin that encapsulates the HASEL actuator, capitalizing on this novel EFKE‐driven mechanism for aquatic locomotion. We conduct comprehensive experiments and analysis on the EFKE utilized to propel the MCR forward against friction. Furthermore, we introduce reconfigurability into the MCR, enhancing its maneuverability, and basically propose two methods for configuring the robot to achieve locomotion with multiple degrees of freedom (DOF). Our reconfigured MCR can locomote in complex trajectories and perform rescue and exploration missions in narrow gap. To improve convenience and reliability, we utilize a commercially available pre‐coated biaxially‐oriented polypropylene (BOPP) membrane in the fabrication of the HASEL actuator, effectively preventing issues like high melting temperature and edge electric breakdown. Additionally, we have optimized the MCR's design parameters and operating voltage to achieve higher motion velocity. Notably, the fully‐soft architecture of the MCR has remarkable robustness and recovery, enabling the robot to continue functioning after enduring significant mechanical impacts.

## 2 Results and Discussion

### 2.1 Principle and Design Rationale

**Figure 1:**
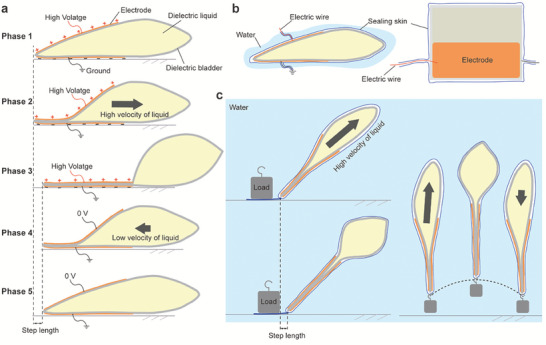
The principle and design of the MCR. a) Principle of the MCR. b) The water‐proof skin. c) Our MCR with the skin can achieve crawling and vertical jumping underwater.

The electrohydraulic actuator here is composed of two flexible electrodes, a deformable dielectric bladder and dielectric liquid enclosed within the bladder, as shown in **Figure** **1**a. The soft dielectric bladder and liquid are sandwiched between two electrodes. Supplied with high voltage (HV), positive and negative charges are induced in the two electrodes respectively, which acts as a charging capacitance. Due to the Maxwell stress generated by the HV, the two electrodes compress the bladder and transfer kinetic energy (EFKE) to the internal liquid. The Maxwell stress *σ_M_* is related to the dielectric permittivity *ε* and the electric field intensity *E*:

(1) $$\sigma_{M}=\frac{1}{2}\;\varepsilon E^{2}$$

To generate unidirectional EFKE, two electrodes are deployed on one side, so that the EFKE can push the MCR toward the other side (Movie S1, Supporting Information). According to Equation (1), the Maxwell stress is highest at the edge of our MCR, so the bladder is zipped by the electrodes (Phases 1 and 2, Figure 1a). The bladder is made from inextensible membranes without bending stiffness, hence it is unable to absorb energy. Most of the EFKE is consumed by the dynamic friction between the ground and the MCR, and the residual EFKE is converted to the gravitational potential energy of the liquid (Phase 3, Figure 1a). Removing the high electrostatic fields, the dielectric liquid flows slowly to its initial position because of its gravitational potential energy (Phases 4 and 5, Figure 1a). The reflux liquid with low kinetic energy cannot propel the MCR. By repeating these processes, our MCR can crawl continuously.

Miniature locomotion robots capable of amphibious mobility have a great advantage in unpredictable field environments and weather. More importantly, a humid or water environment may bring about short‐circuit and electric breakdown that cause huge damage to the electrohydraulic actuator. Hence, we designed a soft water‐proof skin made from the same membrane as the bladder, wrapping and fitting the MCR (Figure 1b). The water‐proof skin allows our MCR to crawl in the water without affecting its mobility since the skin can deform synchronously with the bladder without energy absorption. For underwater crawling, a load is connected to the tail of the MCR (Figure 1c), The load is required to improve the underwater locomotion and to prevent the MCR from floating up since water is denser. Even if the MCR is tilted at a certain angle with the ground by its buoyancy, the EFKE is still able to propel the robot to crawl underwater (Movie S2, Supporting Information). We also illustrated the MCR vertical jumping capability under EFKE actions by reducing the weight of the load (Movie S3, Supporting Information).

### 2.2 Fabrication Based on Pre‐Coated BOPP Film

**Figure 2:**
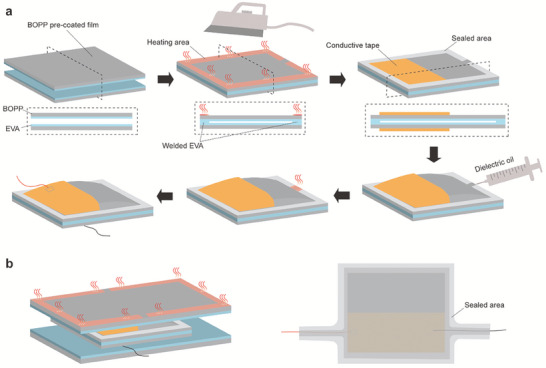
The fabrication process of the MCR. a) The detailed fabrication of the electrohydraulic actuator. b) The fabrication of the water‐proof skin.

To enhance convenience and reliability, we use pre‐coated BOPP film to fabricate the electrohydraulic actuator (MCR). The pre‐coated BOPP film is a composite of a BOPP membrane (16 µm thick) and a layer of ethyl vinyl acetate (EVA) adhesive (9 µm thick). First, we cut the pre‐coated BOPP membrane into 7 cm × 7 cm rectangles, which are stacked on top of each other with the EVA adhesive layers facing each other (**Figure** **2**a). Second, an iron is used to seal the edge of the bladder by melting the EVA adhesive, but leaving a port for liquid injection. We use a 25 µm thick Kapton film to isolate the heating iron from the BOPP film. The iron temperature is about 100 °C and the wielding width of the edge is 1 cm. Third, we attach two conductive tapes (copper tapes with 50 µm thickness and 5 cm width) as electrodes on the BOPP surfaces. Fourth, we inject the dielectric oil into the bladder and seal the port. Lastly, we connect two electric wires to the electrodes. To fabricate the water‐proof skin, we use two pro‐coated BOPP membranes covering the MCR. By a similar path, we seal the skin along the shape of MCR and the electric wires (Figure 2b).

In contrast with previous BOPP‐based fabrication methods,^[^ ^31^ ^]^ the major advantage of our approach is using lower heating temperatures to seal the bladder. Since the melting point of BOPP is ≈170 °C,^[^ ^39^ ^]^ overhigh heating temperature will melt the BOPP layer and the liquidity of melted BOPP material may result in uneven thickness at the welding edge which increases the likelihood of electric breakdown. Furthermore, the commercialized copper tape enables us to easily fabricate electrodes and adjust their position.

### 2.3 Comprehensive Investigation of EFKE

**Figure 3:**
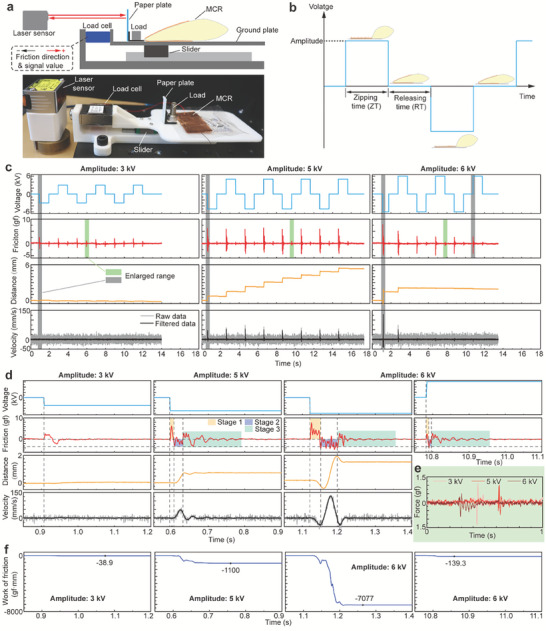
Comprehensive investigation of EFKE. a) The testing setup. b) The applied high voltage. c) The applied voltage, the ground friction force, the motion distance and the motion velocity of the MCR with 3 kV, 5 kV, and 6 kV voltages. d) The transient processes of the enlarged time ranges. e) The ground friction force during the falling edges of applied voltage. f) The work of ground friction force with 3 kV, 5 kV, and 6 kV applied voltages.

According to the principle, the directly‐generated EFKE propels the MCR forward against ground friction. Although it is difficult to directly measure the EFKE, we comprehensively investigate the motion and force of MCR due to EFKE in six related aspects: applied voltage, ground friction force to the MCR, motion distance, instantaneous velocity, fluid reflux, and work of ground friction. We build a testing setup to simulate the real crawling process of the MCR and measure the motion distance and ground friction force as shown in **Figure** **3**a. A 3D‐printed ground plate is connected to a load cell and mounted on a slider to receive vertical load which allows the load cell to only measure the horizontal ground friction force to the MCR. A MCR dragging a load (total weight: 10.9 g) is crawling on the ground plate. We use a laser sensor and a paper plate stuck to the MCR to measure the motion distance of the MCR. To reduce the residual charge, we adopt AC HV as shown in Figure 3b. The down electrode is always grounded, and the up electrode is connected to the HV port which can output a positive or negative HV. The whole operation period consists of zipping time (ZT, the period with HV) and releasing time (RT, the period without HV). We compute the instantaneous velocity *v* of the MCR by differentiation as follows,

(2) $$v\;=\frac{D}{dt}\;$$

where *D* is the motion distance and *dt* is the sampling time interval of 1 ms. The raw velocity data is filtered by a 10‐ms moving average filter. We set the ZT and RT to 1000 ms, and change the amplitude of the voltage from 3 to 6 kV (see the first row of Figure 3c).

The experimental results in Figure 3c indicate that there are fluctuations in the ground friction accompanied by the rising edges of the voltage (see the second row of Figure 3c). The fluctuation peak is the lowest and the step length (motion distance) is maintained to 0 mm when the 3 kV voltage is applied to the MCR (see the third row of Figure 3c). For the 6 kV voltage, the initial two fluctuation peaks of the ground friction are much higher than the following peaks. Similarly, the motion velocity demonstrates only two peaks during the initial two rising edges of the applied voltage. The motion distance of the initial two rising edges of voltage increases obviously, but the step length and the peak of the velocity dramatically decline with time (see the third and fourth rows of Figure 3c). With 5 kV HV, the peak values of the friction and velocity remain almost unchanged. The motion distance continuously steps up with the rising edges of the applied voltage. After the rising edge of voltage, the motion distance keeps temporarily constant and the velocity declines to 0 mm s^−1^ until the next rising edge, thus we further explore the transient process during the rising edge of voltage (see the gray enlarged ranges in Figure 3c,d).

For the 3 kV voltage, we extract the 0.85 to 1.2 s time range (see the first column of Figure 3d). After the voltage rises to ‐3 kV, the direction of ground friction to the MCR is right (see the friction force curve is above 0 gf) because the electrodes are zipping and pushing the liquid to right (Phase 2 in Figure 1a). Then the ground friction turns to left since the EFKE is trying to propel the MCR to right (Phase 3 in Figure 1a). However, the EFKE with 3 kV voltage is insufficient to propel the MCR so that the motion distance is always 0 mm and the ground friction is always static friction. When applying 5 kV voltage, the transient of the ground friction force is divided into 3 stages (Stage 1: orange area, Stage 2: purple area, Stage 1: green area in the second row of Figure 3d). In Stage 1, the direction of ground friction to MCR is right because the electrodes are pushing the liquid to right by the Maxwell stress. At this stage, the MCR does not move and the ground friction force rises up (see the second and third rows of Figure 3d). The impulse from the ground friction force changes the momentum of the inner liquid to increase the speed of the liquid. In Stage 2, the liquid with high kinetic energy (EFKE) propels the MCR to move forward (see the third and fourth rows of Figure 3d), and the ground friction becomes dynamic friction to consume EFKE and the direction turns left. In Stage 3, although the MCR stops, the ground friction keeps fluctuating due to the fluctuation of the inner liquid by the residual EFKE. Finally, the residual EFKE is consumed by the friction between the inner liquid and the friction force remains at 0 gf. For the 6 kV voltage, we present quite different two transient processes (1.05–1.4 s and 10.75–11.1 s in the third column of Figure 3c). In the first transient process (1.05–1.4 s), the impulse from the ground friction force in Stage 1 is much higher than the transient processes with 3 kV and 5 kV (see the third column of Figure 3d), resulting in the EFKE is also the highest. The motion distance of this transient process is the farthest (about 1.5 mm), and the velocity peak value is also the highest (130 mm s^−1^). As for the second transient process (10.75–11.1 s) under 6 kV voltage, the impulse in Stage 1 is extremely low resulting in its negligible EFKE and step length (motion distance) (see the third column of Figure 3c). The reason why the two transient processes with 6 kV voltage are quite different is that too little dielectric fluid flows back between the two electrodes (see Phase 4 in Figure 1a). We then extract the friction force data during the falling edges of applied voltage (see the green enlarged ranges in the second row of Figure 3c). The friction forces in Figure 3e represent the volume of reflux liquid, and the results display that the liquid flows back with 6 kV voltage is the least. Hence, the continuous EFKE is also low due to the insufficient reflux liquid. However, for the initial transient process (1.05–1.4 s), much dielectric liquid is between the two electrodes, so the EFKE is much higher than the others.

The EFKE is consumed by the ground friction force assuming that no friction between the inner liquid. So, the work of ground friction theoretically equals the EFKE. We then compute the work of friction *W* by

(3) $$W\;=\int_{0}^{t}\left(f\cdot v\right)\;dt\;$$

where *f* is the real ground friction force and *t* is the time. The computing results is shown in Figure 3f and illustrate that the work of friction with 5 kV voltage is about −1100 gf mm. The work of friction with 6 kV voltage in the initial transient process is about −7000 gf mm which is almost 50 times more than the work in the following transient process.

### 2.4 Optimization of Crawling Velocity

The crawling velocity is dependent on the EFKE and the operating frequency according to the principle of our MCR. Although higher voltage, in theory, can produce higher EFKE propelling the MCR further, the operating frequency will be restricted by the time it takes for the dielectric liquid to return to its initial position (Phase 4 in Figure 1a). The whole operation period consists of zipping time and releasing time. After removing the HV, the electroadhesion between two electrodes cannot disappear immediately and need a residual time related to the amplitude and duration of HV to recover.^[^ ^40^, ^41^ ^]^ If we reactivate the HV before the sufficient liquid return, the EFKE decreases. This is because little liquid flows back between the two electrodes and the work done by electroadhesion drops down.

**Figure 4:**
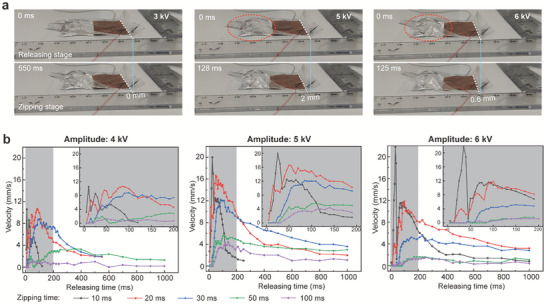
Optimization of crawling velocity. a) The stride with different amplitudes of HV. b) The experimental crawling velocity with various ZT/RT operating patterns and amplitudes of HV.

First, we find the amplitude of the AC HV dramatically influences the stride (Movie S4, Supporting Information). With a 3 kV HV (ZT: 2s; RT: 2s), the MCR is almost stationary, whereas it achieves a 2 mm stride with a 5 kV HV (see **Figure** **4**a). As the HV increases to 6 kV, the stride drops to 0.8 mm because residual electroadhesion hinders the reflux as mentioned (red oval area, Figure 4a). We first have to optimize the ZT and RT that indicate the frequency and duty cycle of the HV, directly for crawling velocity. We control the amplitude of HV to 4, 5, and 6 kV respectively, and measure the velocity of the MCR at different ZT and RT patterns. In this experiment, the ZT is set to five alternative values (10, 20, 30, 50, and 100 ms) and we gradually increase the RT from 5 to 1000 ms. The crawling velocities of all ZTs initially increase and then decrease with increasing RT. As illustrated in Figure 4b, it reaches nearly the maximum value within 200 ms of RT, so we conducted more detailed tests within this range (gray areas in Figure 4b). With 10 ms ZT, the velocity of all HV amplitudes dramatically fluctuates within 50 ms of RT, and then reaches a relatively high and stable plateau. With 20 ms ZT, the velocity increases with fluctuation initially and then reaches its maximum value which is higher than that plateau of 10 ms ZT. After that, the crawling velocity slowly decreases with RT. With 30 ms ZT, the velocity rises smoothly with RT in the beginning, followed by a gradual descent too. The velocity of 50 and 100 ms ZT is much lower than others within 200 ms of RT. Based on the specific HV amplitudes and ZTs, we can choose corresponding RT values to achieve high and stable crawling velocity. According to the above criteria, we selected optimized ZT/RT operating patterns for different amplitudes of HV: 10/60, 20/80, and 30/120 ms for 4 and 5 kV HV; 10/80, 20/120, and 30/180 ms for 6 kV HV.

**Figure 5:**
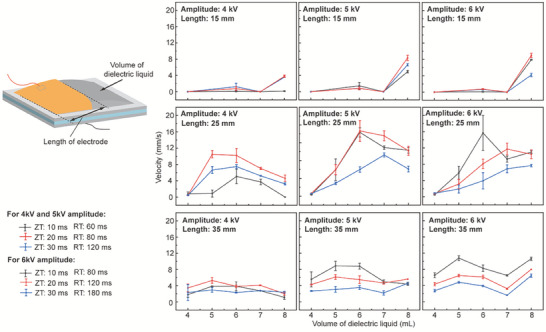
The MCR's velocity with alternative design parameters and amplitudes of HV.

Based on the selected ZT/RT patterns, we proceed to optimize the amplitude of HV, the length of the electrode and the volume of the dielectric liquid of the MCR (**Figure** **5**). We fabricated MCRs with various length of electrodes at 15, 25, and 35 mm at 5 cm widths. In addition, various volumes (4, 5, 6, 7, and 8 mL) of dielectric oil were explored. With a 15 mm length of electrode, all crawling velocities under all HV patterns are <2 mm s^−1^, if the volume is under 7 mL. When the volume surpasses 7 mL, the velocity is from 3 to 8 mm s^−1^. With a 25 mm length of electrode, MCR's average velocity improved significantly more than others. They exhibited the highest crawling speed of 16 mm s^−1^ with 5 kV HV (ZT/RT: 20/80 and 10/60 ms) and 6 mL oil. With a 35 mm length of electrode, the velocities fall between of 15 mm length and 25 mm length data and show no obvious trends. Based on these experimental data, we selected the 25 mm long electrode, the 6 mL dielectric oil and the 5 kV amplitude of HV. The optimized MCR weighs 6.3 g, and it is 5 cm long, 5 cm wide, and 6 mm high (maximum value in operation).

**Figure 6:**
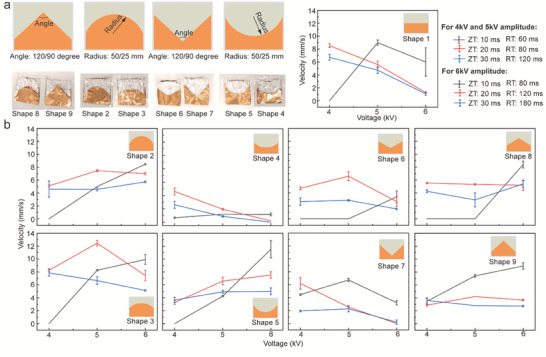
The MCR's velocity with the tailored shape of electrodes. a) The customized shape of electrodes and the velocity of MCR with rectangle electrodes. b) The velocity of MCR with other various shapes of electrodes.

Due to the uneven distribution of inner dielectric liquid in the width direction of the electrodes, we then optimize the shape of the electrodes. The distance between the two electrodes at the middle area is higher than it at the edge area due to the soft electrodes and bladder, resulting in the unevenly generated EFKE. Here, we change the length of electrodes in the width direction from the constant value (rectangle, Shape 1 in **Figure** **6**a) to the variable value (Shape 2: convex circle with 25 mm radius, Shape 3: convex circle with 50 mm radius, Shape 4: concave circle with 50 mm radius, Shape 5: concave circle with 25 mm radius, Shape 6: concave triangle with 120° angle, Shape 7: concave triangle with 90° angle, Shape 8: convex triangle with 120° angle, Shape 9: convex triangle with 90° angle, in Figure 6a). Shapes 2–8 keep the same electrode area (1250 mm^2^) as Shape 1, and all the MCRs have the 6 mL dielectric liquid. We test the average crawling velocity of MCR with the same applied voltage in Figure 5 and the experimental results of the customized shapes of electrodes are shown in Figure 6b. The results demonstrate that almost all the velocities of concave shapes (Shapes 4–7) are lower than the rectangle electrodes (Shape 1). Among other shapes, the MCRs with convex circle electrodes have relatively higher crawling velocities. Especially the convex circle shape with a 50 mm radius, its maximum velocity reaches 12 mm s^−1^ with 5 kV and 20/80 ms ZT/RT operating pattern and the other velocities of this shape are also higher than the rectangle shape.

### 2.5 Design Reconfiguration for 2‐DoF Locomotion

**Figure 7:**
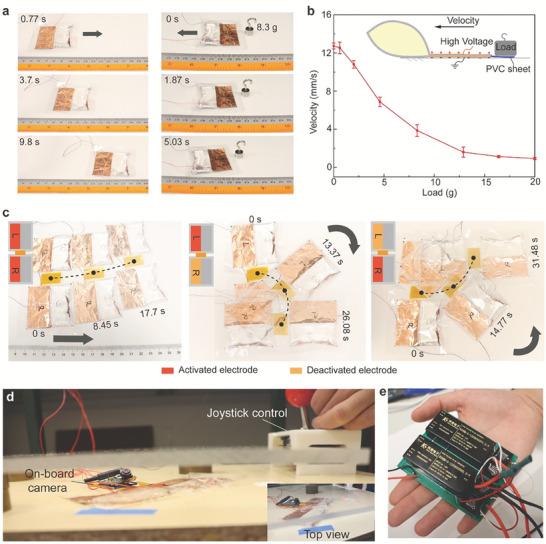
The single‐DoF MCR and 2‐DoF MCR. a) The single‐DoF MCR drags a load. b) The crawling velocities with different loads. c) The reconfigured 2‐DoF MCR is composed of two MCRs. d) The reconfigured 2‐DoF MCR manually controlled by a joystick can search in a narrow gap. e) A portable two‐channel AC high voltage power supply for MCR.

Based on the above‐optimized design and operating HV, the MCR can crawl at a fast speed (Movie S5, Supporting Information). Even dragging a load, it can also achieve effective locomotion (**Figure** **7**a). We tested the crawling velocity of the MCR with different loads from 0 to 20 g (317% of the weight of MCR). To facilitate electrode manufacturing, we still use the rectangle electrodes of MCR. The results (Figure 7b) demonstrated that the velocity decreases with the load from 12.7 to 0.94 mm s^−1^.

To improve its maneuverability, we reconfigured the MCR to enable 2‐DoF locomotion in a plane. One approach is to connect two MCRs in parallel. By applying the same HV in the two MCRs, the 2‐DoF robot moves forward (Figure 7c). If only the left MCR is activated, the robot can turn right at 3.46° s^−1^. Reversely, it turns left at 2.86° s^−1^ with the activated right MCR (Movie S6, Supporting Information). To demonstrate its application potential in narrow space, we connect an on‐board camera to the 2‐DoF MCR and also develop a hardware system to control the MCR by manual operating a joystick (see Figure 7d).

In experimental stage, a 24.9 kg heavy and large (dimensions: 279 × 482 × 654 mm) off‐board high voltage device (Trek 10/40, Advanced Energy) is used to power our MCR as shown in Figure S1, Supporting Information, which seriously limited practicality and autonomy of our MCR for real‐world application, such as field search and rescue. To address this limitation, we have developed a lightweight (175 g), portable and compact (dimensions: 25 × 74 × 82 mm) high voltage power supply for our MCR (see Figure 7e and Figure S2, Supporting Information). The HV power could output 2 channels of AC HV with different period and duty so that it can allow 2 MCRs to move at different speeds.

**Figure 8:**
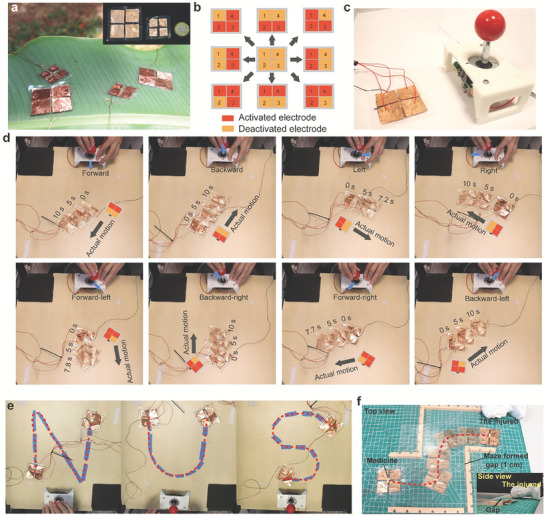
The 4‐electrode‐MCR and its motion. a) The 4‐electrode‐MCR. b) The four crawling directions of the 4‐electrode‐MCR. c) The 4‐electrode‐MCR is manually controlled by a joystick. d) The 4‐electrode‐MCR can move in 8 directions. e) Our 4‐electrode‐MCR can be controlled to follow three challenging paths like “N,” “U,” and “S.” f) The 4‐electrode‐MCR passes through a winding gap and delivers medicine to the injured.

Another approach is to reconfigure the distribution of electrodes on the MCR. Here, we split the MCR into four areas and deploy four corresponding pairs of electrodes (**Figure** **8**a). The MCR can generate EFKE in 8 directions and enable the MCR to crawl multi‐directionally by activating different electrodes as shown in Figure 8b,d and Movie S7, Supporting Information. By manually controlling our 4‐electrode‐MCR with a joystick (see Figure 8c), the MCR can achieve agile motion for complex missions and follow some complex trajectories (see Movie S7, Supporting Information and Figure 8e). Compared with the first approach, the 4‐electrode‐MCR requires only one electrohydraulic actuator making it more compact. Therefore, it has advantages, especially in confined spaces for rescue tasks. To show its potential, we controlled the 4‐electrode‐MCR transporting medication while passing through a winding gap (1 cm) to the injured (Figure 8f and Movie S8, Supporting Information).

### 2.6 Underwater Locomotion

**Figure 9:**
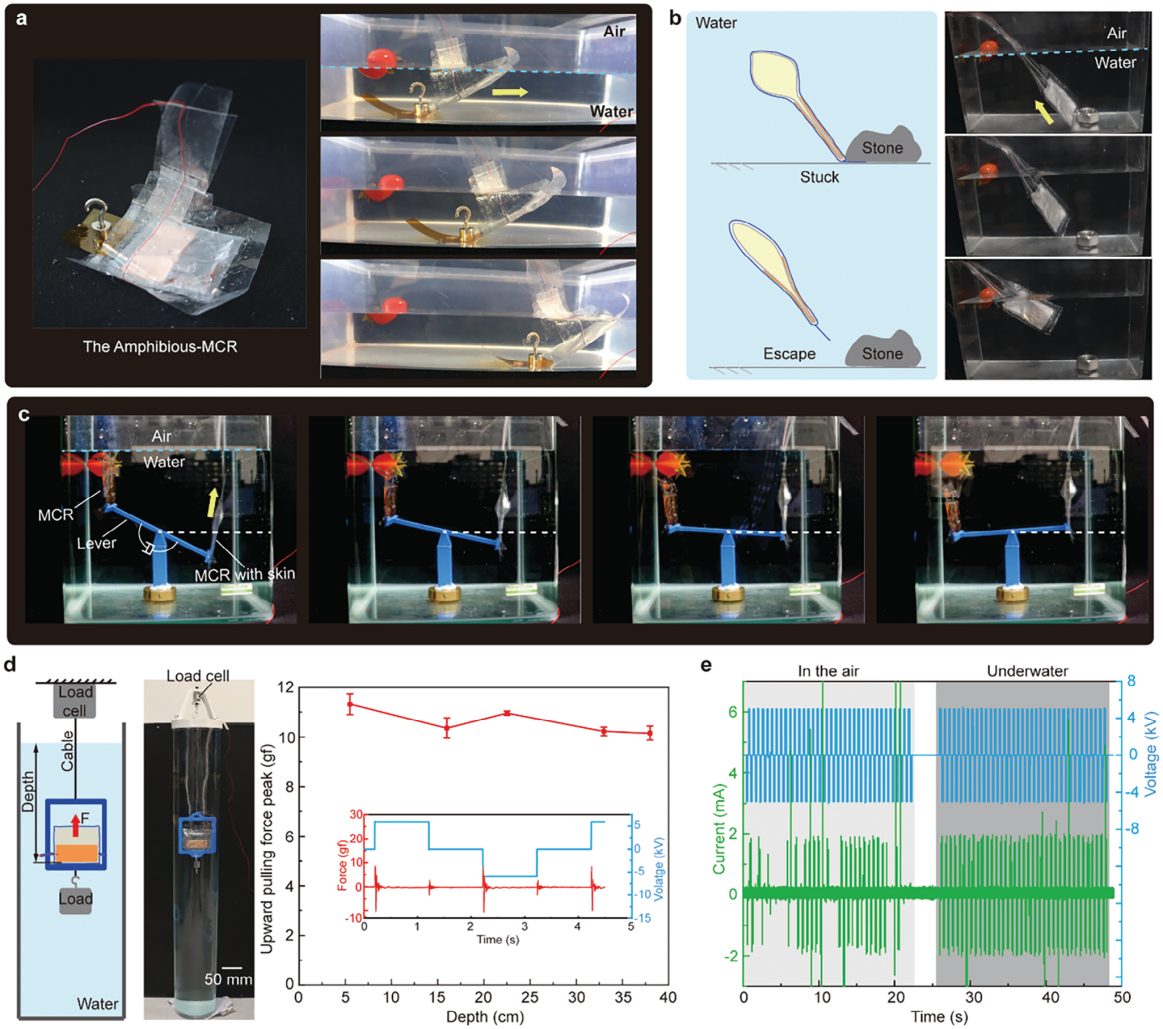
Underwater locomotion of the MCR. a) The MCR with water‐proof skin crawls underwater dragging a load. b) The MCR escapes from being stuck. c) The MCR rotates a lever. d) The influence of water depth on EFKE. e) The output current of HV supply.

With the waterproof skin, the MCR can crawl underwater (**Figure** **9**a and Movie S2, Supporting Information). With principle shown in Figure 1c, an 8.3 g weight was connected to the MCR tail allowing it to sink to the bottom (4 cm depth). The robot dragging the weight crawls step by step powered by EFKE. In addition, we showcased the ability to escape a larger load (63 g) with repeated generation of EFKE, when the MCR tail was pinned down (Figure 9b). Additionally, the underwater EFKE can be utilized to actuate other mechanisms under water. To simulate a scenario that our MCR propels with a load and swims upward, we introduced an application for our MCR to rotate an underwater lever. Here, we built an underwater lever with the pivot point in the middle allowing for free rotation. One end of the lever was connected to the water‐proof MCR (right), and the other end was connected to another MCR with water‐proof skin (left) for balance. By powering the water‐proof MCR, the generated upward EFKE repeatedly struck the bar and gradually rotated the lever (Figure 9c and Movie S9, Supporting Information). This application displays our MCR can achieve 3D locomotion in underwater environment.

We further explored the influence of water depth on the underwater EFKE. The MCR with skin was fixed on a rigid frame that is connected to a load cell via a cable (Figure 9d). The rigid frame together with the MCR was immersed in the water connect to a 100 g weight pulling the frame down to straighten the cable. The load cell can measure the actual force variation due to the underwater EFKE. We change the immersing depth from 5.5 to 38 cm and record the variation peak of the force which also implicitly indicates the EFKE. The results (Figure 9d) show that water depth cannot influence the EFKE. The whole volume of the MCR keeps constant owing to the liquid incompressibility. Assuming the pressure on the surface of the robot is equal everywhere, the robot hardly transfers energy to the water. Therefore, the water depth cannot influence the EFKE of the MCR with water‐proof skin. Furthermore, we also compared the output current of the HV supply between the in‐air and underwater MCRs. The water environment influences the operating current of the MCR (Figure 9e). The current peaks of underwater MCR were denser than those in the air likely due to the water increasing the capacitance. We then compute the average power consumption of the MCR based on the input current and voltage in Figure 9e. The energy cost of the transportation for the soft robot varies with environment. The average power of the robot for land locomotion is about 2.2 mW, while the power is 5.8 mW for underwater operation.

### 2.7 Robustness, Recovery and Durability of MCR

**Figure 10:**
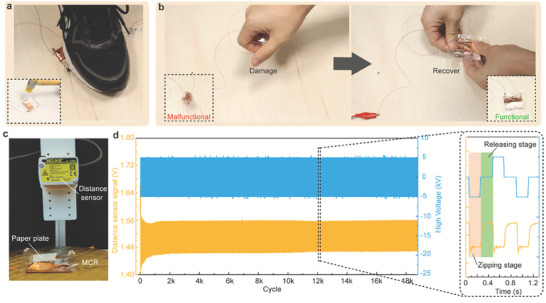
Robustness, recovery, and durability test. a) Step on the MCR with human feet and hammer impact on the MCR. b) Recover the MCR after damage. c) The platform for durability test. d) The durability test results.

Benefiting from the fully‐soft body, our MCR possesses high robustness. It can work normally despite being stepped on (**Figure** **10**a and Movie S10, Supporting Information) by a human. Even hammer impact cannot damage the structure of MCR due to its highly deformable components (liquid and films). In addition, our MCR has high recovery stemming from its compliant components (electrodes, the dielectric bladder, and internal liquid). We intentionally knead the MCR trying to cause a malfunction. In spite of that, a simple stretch can recover its function (Figure 10b and Movie S11, Supporting Information). The durability of MCR was also tested by the testing platform shown in Figure 10c. We adhered a piece of paper on top of the MCR and used a laser displacement sensor to measure the deformation of electrohydraulic actuator. A 5 kV AC HV (ZT/RT: 200/200 ms) powered the electrohydraulic actuator. Our MCR can run continuously for about 19k cycles without attenuation (more than 2 h).

## 3 Conclusion

In this work, we designed a fully‐soft miniature crawling robot base on the directly‐generated EFKE by electrohydraulic actuator. The MCR consisted of only an electrohydraulic actuator which was made from pre‐coated BOPP membranes with lower heating temperature and reliable performance. After comprehensively optimizing the operating HV, the volume of dielectric liquid, the shape and size of electrodes, our MCR achieved 16 mm s^−1^ crawling speed (0.32 body length per second). The optimized MCR weighs 6.3 g and has a 50 × 50 × 6 mm dimension, which is still scalable. We also proposed two effective ways to reconfigure our MCR for 2‐DoF locomotion and complex trajectory tracking which has immense potential in rescue and delivery operations. To extend its environmental adaptability, we designed a soft water‐proof skin fitted onto the MCR which allows our MCR to locomote and actuate underwater. The fully‐soft and deformable structure gave the robot favorable robustness and recovery that preserved the robot's functionality. Moreover, the MCR exhibited high durability verified from our experiments.

Compared with the groundbreaking HASEL actuator,^[^ ^30^ ^]^ our work mainly focuses on the soft locomotion robot directly powered by EFKE. For robotic locomotion application, besides the basic actuator, other factors also significantly influence the performance such as the principle, design, and control. The scientific contributions of this paper are as follows. First, we innovatively explore the principle of propelling an amphibious fully‐soft miniature crawling robot. Second, based on the novel crawling principle, we systemically optimized the operating voltage (amplitude, period, and duty ratio) and the design parameters (volume of dielectric liquid, length of electrodes, and shape of electrodes) to enhance crawling speed. Third, we comprehensively investigated the EFKE in six aspects: applied voltage, ground friction force to the MCR, motion distance, instantaneous velocity, fluid reflux, and work of ground friction. Last, we additionally reconfigured the crawling robot for remarkable 2‐DOF maneuverability.

In contrast with other soft miniature crawling robots (**Table** **1**), our MCR with full softness can achieve relatively high crawling velocity. The EFKE‐driven mechanism considerably improves the crawling speed, almost 290 times that of the other MCR actuated by electrohydraulic (HASEL) actuators.^[^ ^36^ ^]^ Most other MCRs require rigid leg or body architectures, which limits the softness of the robots. Moreover, our soft MCR equipped with soft water‐proof skin has a certain amphibious ability. Regarding the reliance on tethered operation, we think this is a potential limitation of the study. An onboard driving and control circuit can extremely increase practicality and autonomy of our MCR. However, it will also reduce the softness and compliance of our MCR due to the rigid electronic components and PCB (Printed circuit board). More importantly, based on the current research status, it is very difficult to develop a lightweight (<10 g) onboard circuit enabling to output and switch a up to 6 kV high voltage. Among those soft miniature crawling robots that rely on high voltage technology (other works^[^ ^24^, ^36^, ^37^, ^42^ ^]^ and our work, see Table 1), only one MCR^[^ ^24^ ^]^ achieved untethered operation due to its relatively low voltage (<500 V).

**Table 1:** Comparison with other soft miniature crawling robots.

Soft crawling robot	Actuation type	Softness	Average velocity [BL s^−1^]	Body length [mm]	Body weight [g]	Amphibious ability	Untethered
[36]	Electrohydraulic + peristaltic mechanism	Full	0.0011	125	8	No	No
[37]	Electrohydraulic + inchworm mechanism	Part	0.077	140	—	No	No
[42]	Electroadhesion	Part	0.57	46	0.23	No	No
[43]	IPMCs^a)^	Part	0.016/0.15^b)^	28	2.7	High	Yes
[24]	DEA	Part	0.3	40	1	No	Yes
[44]	SMA	Part	0.56	57	25	No	Yes
[19]	Pneumatics	Full	0.047	60	10	High	No
This work	Electrohydraulic + EFKE	Full	0.32	50	6.3	Medium^c)^	No

^a)^ Ionic polymer–metal composites actuator;

^b)^ 0.016 is for the crawling state and 0.15 is for the swimming state;

^c)^ The MCR equipped with water‐proof skin.

Future work involves further exploring the amphibious ability, such as seamlessly transitioning between water and land and improving the swimming ability by integrating soft paddles onto the MCR. New dielectric materials can also be explored with the aim of reducing the operating voltage. In addition, an untethered MCR can be explored using a compact and lightweight power on‐board circuit.

## 4 Experimental Section

### Testing Circuit System

The MCR was powered by a high voltage supply (Trek 10/40, Advanced Energy) via 0.3 mm diameter Teflon electric wires and 0.8 mm 30AWG silicone wires in Figures 6 and 8. The high voltage supply's ports were connected to a multifunction DAQ device (USB‐6211, National Instruments). The DAQ device was programmed by LabVIEW 2018. For the experiment in Figure 3, the load cell (JLBS‐M2, ZWJN) were connected to the DAQ through a micro‐signal amplifier (AMP01, HUAXIN) and the laser sensor (BL‐100NMZ, BOJKE) was directly connected to the DAQ (see Figure S1, Supporting Information). The A/D sampling rate and the D/A outputting rate of the DAQ were set to 1000 Hz. The ZT, RT, and the amplitude of HV were controlled by regulating the parameters in the LabVIEW procedure.

### Portable AC HV Power Supply

A positive DC HV power supply (KDHM‐G‐12S8000P0.2‐V, Xian Corso Electronic Technology, Co. Ltd.) and a negative DC HV power supply (KDHM‐G‐12S8000N0.2‐V, Xian Corso Electronic Technology, Co. Ltd.) were used to generate two DC HV with different polarities (See Figure S2 and Table S1, Supporting Information). The amplitudes of the two DC HV power supply were adjusted by a microcontroller (Arduini Nano, Arduino) to a same value. Then a “half bridge circuit” made from two HV optocouplers (OPTO‐150, HVM Technology, Inc.) modulated the two DC HV with different polarities to AC HV. Two half bridge circuits and generate were integrated into the portable AC HV power supply to produce 2 channel AC HV with same amplitude but independent period and duty ratio.

### Joystick Control System

A microcontroller (Mega 2560, Arduino) was used to read the state of the joystick which was composed of four switches and recognized the human intended moving direction. Then the microcontroller independently controlled the ON/OFF state of four HV relays (CRSTHV‐20KV‐A, CRSTRELAY) through the MOSFET circuit (see Figure S3, Supporting Information). The 4 HV relays were corresponding to four independent electrodes.

### Velocity Test

To test the crawling velocity, except the instantaneous velocity in Figure 3, the total operating time was set to 5 s, and the motion distance was measured by a rule to calculate the average crawling velocity. The slow‐motion videos were shot by a cellphone (Realme GT 2Pro, OPPO).

## Conflict of Interest

The authors declare no conflict of interest.

## Supporting information

Supporting Information

Supplemental Movie 1

Supplemental Movie 2

Supplemental Movie 3

Supplemental Movie 4

Supplemental Movie 5

Supplemental Movie 6

Supplemental Movie 7

Supplemental Movie 8

Supplemental Movie 9

Supplemental Movie 10

Supplemental Movie 11

## Data Availability

The data that support the findings of this study are available from the corresponding author upon reasonable request.
