# Supplementary material for: An Amphibious Fully‐Soft Centimeter‐Scale Miniature Crawling Robot Powered by Electrohydraulic Fluid Kinetic Energy
Source: Adv Sci (Weinh). 2024 Feb 1;11(14):2308033. doi: 10.1002/advs.202308033 (PMC11005735; doi:10.1002/advs.202308033)
Supplement: Supplementary file 1 — Supporting Information [file ADVS-11-2308033-s008.pdf]

ADVANCED SCIENCE
Open Access

Supporting Information

for *Adv. Sci.*, DOI 10.1002/advs.202308033

An Amphibious Fully-Soft Centimeter-Scale Miniature Crawling Robot Powered by Electrohydraulic Fluid Kinetic Energy

Quan Xiong, Xuanyi Zhou, Dannuo Li, Jonathan William Ambrose and Raye Chen-Hua Yeow**

Supplementary Materials for
An Amphibious Fully-Soft Miniature Crawling Robot Powered by Electrohydraulic Fluid Kinetic Energy

Quan Xiong, Xuanyi Zhou, Dannuo Li, Jonathan William Ambrose, Raye Chen-Hua Yeow*

*Corresponding author. Email: rayeow@nus.edu.sg

This PDF file includes:

Figures S1 to S3
Table S1

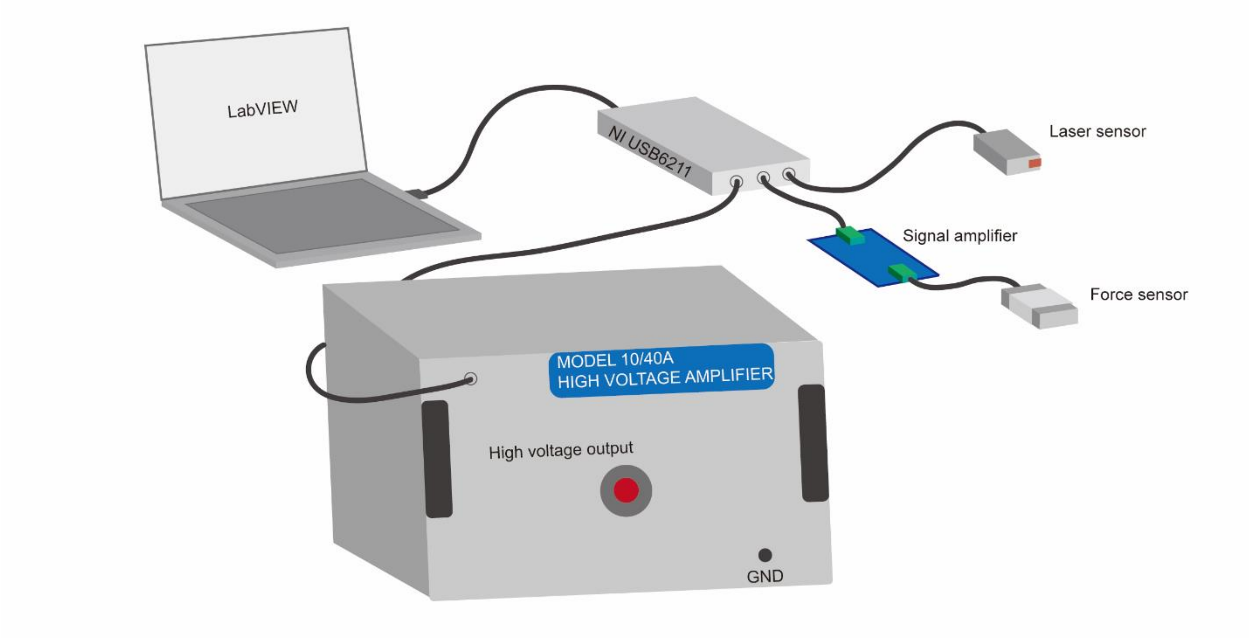

Figure S1. The circuit system. The load cell is connected to the NI DAQ through a micro-signal amplifier and the laser sensor is directly connect to the DAQ.

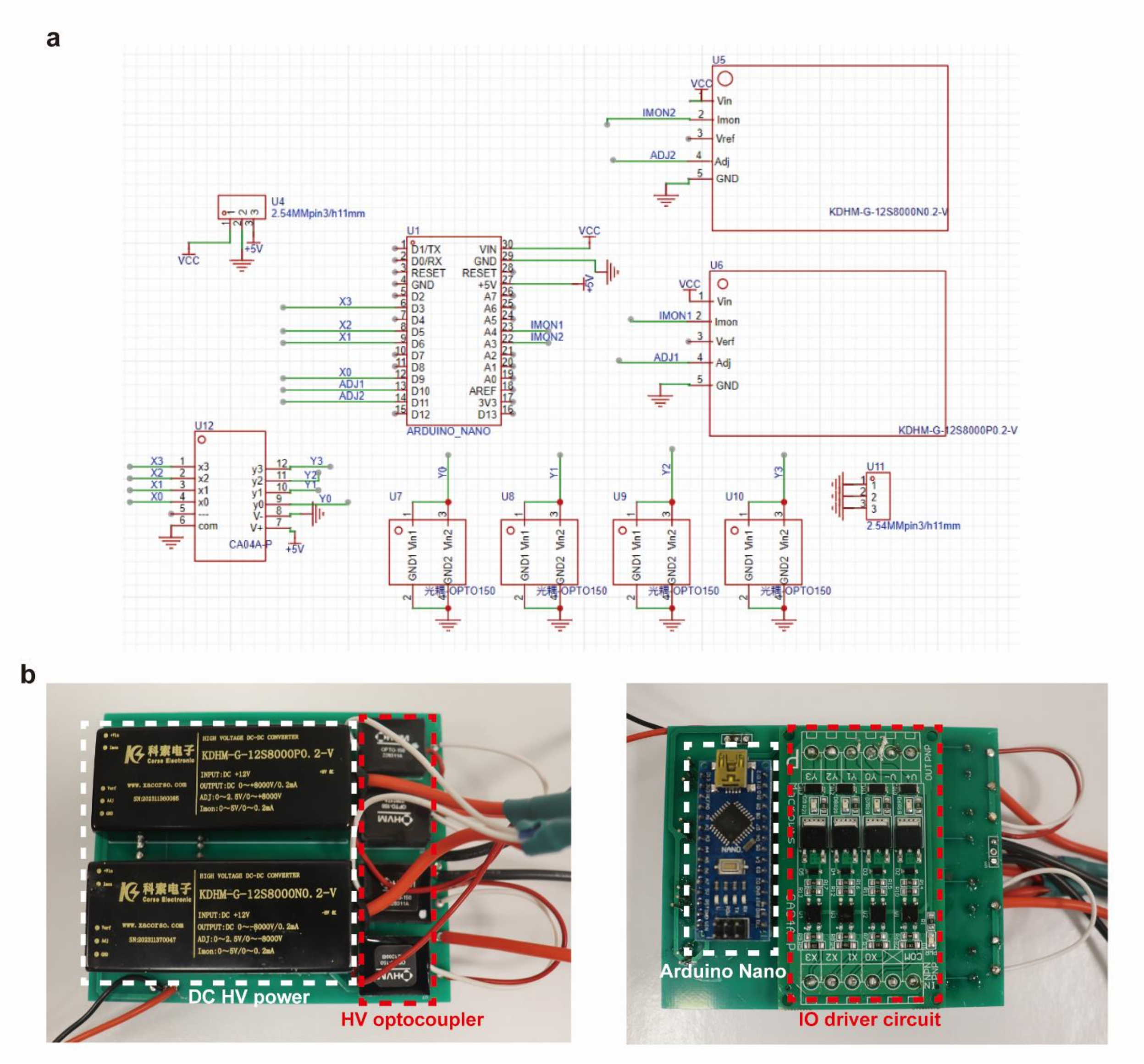

Figure S2. A portable two-channel AC high voltage power supply for MCR. (a) The schematic circuit diagram. (b) The front view and back view of the portable AC high voltage power supply.

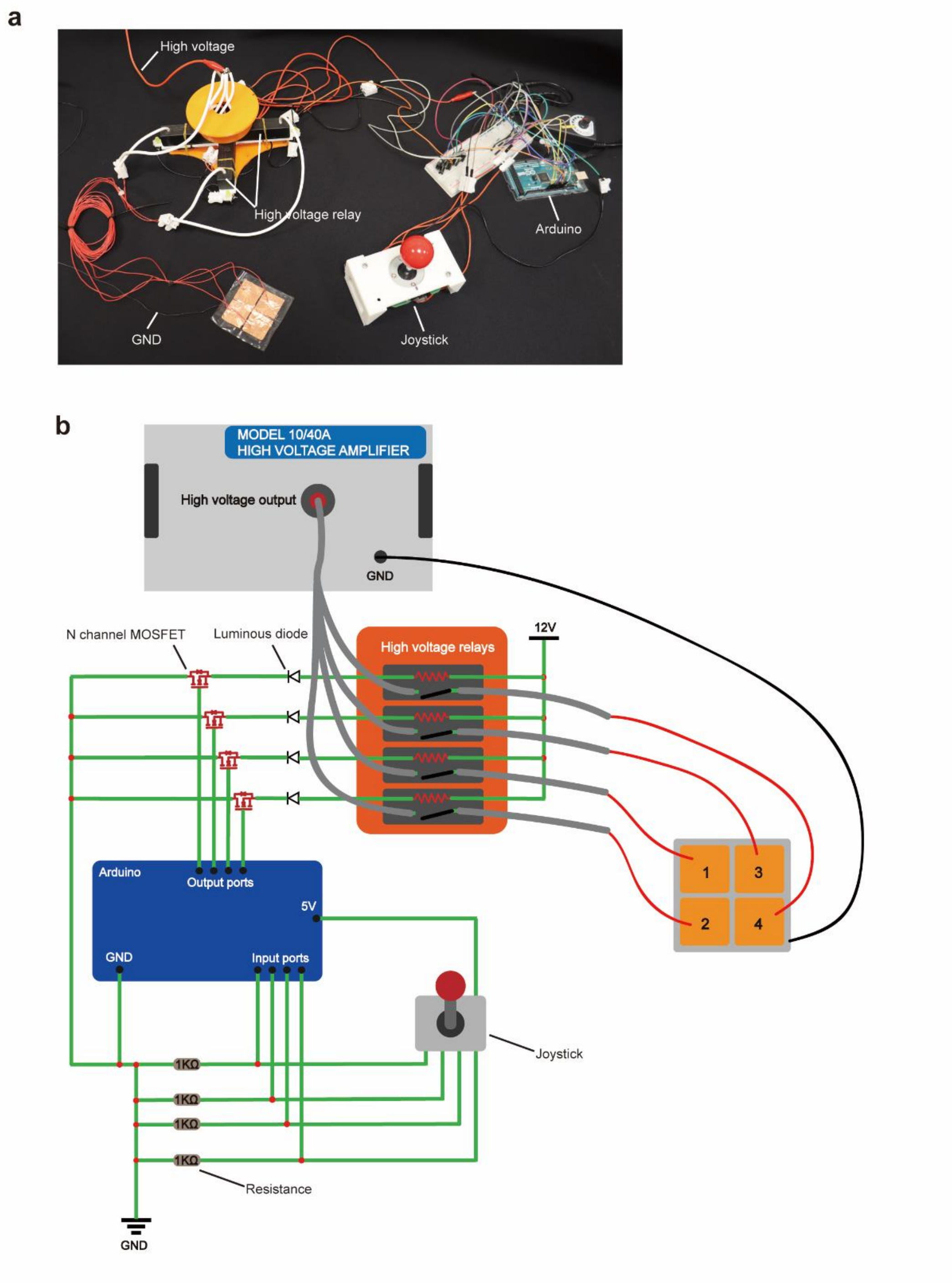

Figure S3. Joystick control system. (a) The assembled joystick control system for a 4-electrode-MCR. (b) The circuit design of the joystick control system.

Table S1 BOM (Bill of Material) of the portable two-channel AC high voltage power supply

Components	Model	Number	Manufacturer
High voltage power supply	KDHM-G-12S8000P0.2-V	1	Xian Corso Electronic Technology, Co. Ltd.
High voltage power supply	KDHM-G-12S8000N0.2-V	1	Xian Corso Electronic Technology
Micro controller	Arduino Nano	1	Arduino
High voltage optocoupler	OPTP-150	4	HVM Technology, Inc.
IO driver circuit	CAO4A-P	1	Macrowis
